# Vital Signs: HIV Diagnosis, Care, and Treatment Among Persons Living with HIV — United States, 2011

**Published:** 2014-11-28

**Authors:** Heather Bradley, H. Irene Hall, Richard J. Wolitski, Michelle M. Van Handel, Amy E. Stone, Michael LaFlam, Jacek Skarbinski, Darrel H. Higa, Joseph Prejean, Emma L. Frazier, Roshni Patel, Ping Huang, Qian An, Ruiguang Song, Tian Tang, Linda A. Valleroy

**Affiliations:** 1 Division of HIV/AIDS Prevention, National Center for HIV/AIDS, Viral Hepatitis, STD, and TB Prevention, CDC

## Abstract

**Background:**

Infection with human immunodeficiency virus (HIV), if untreated, leads to acquired immunodeficiency syndrome (AIDS) and premature death. However, a continuum of services including HIV testing, HIV medical care, and antiretroviral therapy (ART) can lead to viral suppression, improved health and survival of persons infected with HIV, and prevention of HIV transmission.

**Methods:**

CDC used data from the National HIV Surveillance System and the Medical Monitoring Project to estimate the percentages of persons living with HIV infection, diagnosed with HIV infection, linked to HIV medical care, engaged in HIV medical care, prescribed ART, and virally suppressed in the United States during 2011.

**Results:**

In 2011, an estimated 1.2 million persons were living with HIV infection in the United States; an estimated 86% were diagnosed with HIV, 40% were engaged in HIV medical care, 37% were prescribed ART, and 30% achieved viral suppression. The prevalence of viral suppression was significantly lower among persons aged 18–24 years (13%), 25–34 years (23%), and 35–44 years (27%) compared with those aged ≥65 years (37%).

**Conclusions:**

A comprehensive continuum of services is needed to ensure that all persons living with HIV infection receive the HIV care and treatment needed to achieve viral suppression. Improvements are needed across the HIV care continuum to protect the health of persons living with HIV, reduce HIV transmission, and reach prevention and care goals.

**Implications for public health practice:**

State and local health departments, community-based organizations, and health care providers play essential roles in improving outcomes on the HIV care continuum that increase survival among persons living with HIV and prevent new HIV infections. The greatest opportunities for increasing the percentage of persons with a suppressed viral load are reducing undiagnosed HIV infections and increasing the percentage of persons living with HIV who are engaged in care.

## Introduction

In the United States, an estimated 1.2 million persons are living with human immunodeficiency virus (HIV), a serious infection that, if untreated, leads to illness and premature death. Persons living with HIV who use antiretroviral therapy (ART) and achieve very low levels of the virus (suppressed viral load) can have a nearly normal life expectancy (1) and have very low risk for transmitting HIV to others (2). However, each year in the United States, nearly 50,000 persons become infected with HIV (3). Each step along the HIV care continuum (HIV diagnosis, prompt and sustained HIV medical care, and ART) is essential for achieving a suppressed viral load.

To accelerate progress toward reaching the goals of the National HIV/AIDS Strategy (NHAS), which include reducing new HIV infections, improving health outcomes among persons living with HIV, and reducing HIV-related disparities, the President issued an executive order in July 2013 directing federal agencies to improve outcomes along the HIV care continuum, with the goal of increasing viral suppression among persons living with HIV. This report estimates the number of persons living with HIV who received selected services along the HIV care continuum in the United States and the overall percentage of persons with suppressed viral load.

## Methods

Data reported through December 2013 to the National HIV Surveillance System (NHSS) from 50 states and the District of Columbia were used to estimate the number of persons living with HIV infection and the number living with diagnosed HIV by year-end 2011. The number of persons living with HIV infection (prevalence) was estimated as previously described (4). NHSS data from 19 jurisdictions (18 states and the District of Columbia)* with complete laboratory reporting were used to estimate linkage to HIV medical care. Linkage to HIV medical care was defined as one or more documented viral load or CD4+ T-lymphocyte (CD4+) count test within 3 months after HIV diagnosis and was estimated among persons aged ≥13 years newly diagnosed with HIV in 2011.

Data from the Medical Monitoring Project were used to estimate the number of persons aged ≥18 years with HIV engaged in care, prescribed ART, and with a suppressed viral load (5). Numbers are weighted, nationally representative population estimates from a complex sample survey of persons in HIV medical care in the United States. Being engaged in care was defined as having had an HIV medical care visit during the survey’s sampling period of January–April 2011. ART was defined as documentation in the medical record of an ART prescription during the 12 months preceding interview. Viral suppression was defined as documentation in the medical record of viral load <200 copies/mL at last viral load test in the 12 months preceding interview. Statistical testing of differences between groups was conducted using the delta method (6).

## Findings

**FIGURE 1:**
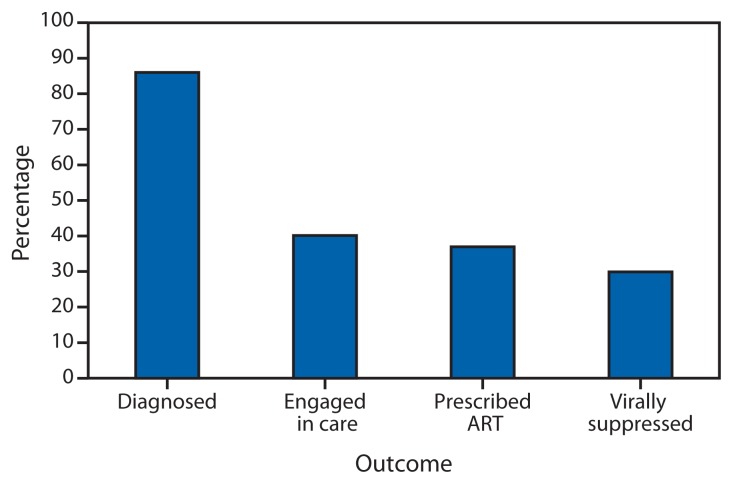
Estimated percentage of persons living with HIV infection,^*^ by outcome along the HIV care continuum — United States, 2011 **Abbreviations:** HIV = human immunodeficiency virus; ART = antiretroviral therapy. ^*^ N = 1,201,100.

In 2011, an estimated 1.2 million persons were living with HIV infection in the United States; an estimated 86% were diagnosed with HIV, 40% were engaged in HIV medical care, 37% were prescribed ART, and 30% achieved viral suppression (Figure 1).

The prevalence of viral suppression was significantly lower among persons aged 18–24 years (13%), 25–34 years (23%), and 35–44 years (27%) compared with those aged ≥65 years (37%). (Table 1). An estimated 28% of blacks achieved viral suppression, compared with 32% of whites, a difference that was not statistically significant.

Of 15,449 persons newly diagnosed with HIV in the 19 surveillance areas in 2011, 80% were linked to HIV medical care within 3 months. Linkage to care was lowest among persons aged 13–24 years (73%) and blacks (76%) (Table 2).

**FIGURE 2:**
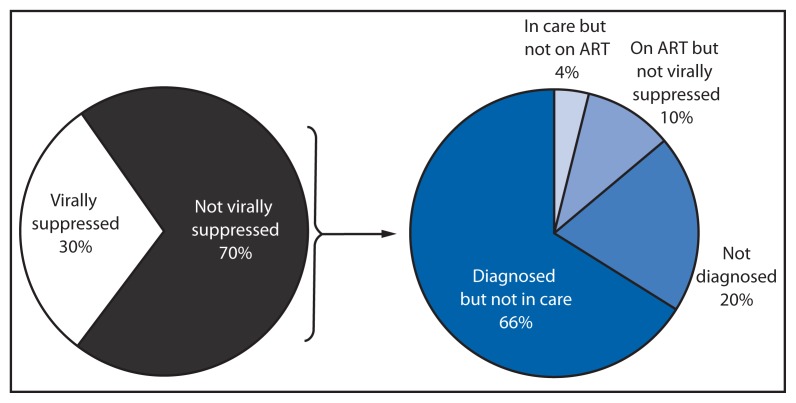
Estimated percentage of persons living with HIV infection,^*^ by viral suppression status, and estimated percentage of persons living with HIV infection who were not virally suppressed,^†^ by diagnosis and treatment status — United States, 2011 **Abbreviations:** HIV = human immunodeficiency virus; ART = antiretroviral therapy. ^*^ N = 1,201,100. ^†^ N = 839,336.

Of the estimated 1.2 million persons living with HIV, an estimated 839,336 (70%) had not achieved viral suppression. Of these 839,336, an estimated 20% had never been diagnosed with HIV, 66% had been diagnosed but were not engaged in HIV medical care, 4% were in HIV medical care but had not been prescribed ART, and 10% had been prescribed ART but had not achieved viral suppression (Figure 2).

### Discussion

Of persons living with HIV in the United States in 2011, 30% achieved viral suppression. This percentage was relatively stable from 2009 (26%) to 2011 (4). Improvements are needed across the HIV care continuum to protect the health of persons living with HIV, reduce HIV transmission, and reach national prevention and care goals. The greatest opportunities for increasing the percentage of persons with a suppressed viral load are reducing undiagnosed HIV infections and increasing the percentage of persons living with HIV who are engaged in care.

HIV diagnosis is the entry point of the HIV care continuum. All adolescents and adults should be tested for HIV infection at least once. Pregnant women should be tested, including those presenting at labor with an unknown HIV status. Persons at increased risk for HIV infection should be tested at least annually, including men who have sex with men (MSM), persons who inject drugs, and persons presenting for sexually transmitted disease testing (7,8).

Persons living with HIV must receive HIV medical care to benefit from being prescribed ART, becoming virally suppressed, and receiving prevention counseling to reduce risk behaviors. Approximately 66% of persons who did not have a suppressed viral load were diagnosed with HIV but not engaged in HIV medical care. Thus, interventions that increase the likelihood that such persons will seek and receive ongoing medical care are essential. Strategies include strengths-based case management (i.e., encouraging patients to identify and use internal strengths and assets to overcome obstacles), provider notification systems, co-located medical and support services, clinic materials promoting engagement in care, clinic staff who have expertise serving affected subpopulations (e.g., youths and gay men), and appointment scheduling (9). Patient navigation and outreach services might also be helpful (10).

To prevent deterioration of immune function, prolong life, and decrease transmission risk, all persons diagnosed with HIV should receive medical care and be offered ART as soon as possible after diagnosis with HIV infection, regardless of CD4+ count or HIV viral load (11). HIV medical care and treatment have multiple benefits. Most persons in HIV medical care are prescribed ART (92%) and achieve viral suppression (76%). Persons who are diagnosed with HIV at age 20 years and initiate ART immediately and consistently throughout their lives can expect to live an additional 51 years, which approaches the life expectancy of a person aged 20 years in the general population (1). Early ART has been shown to reduce the likelihood of sexual transmission of HIV by 96% (2). However, one in five newly diagnosed persons was not linked to care within 3 months, missing an important opportunity to receive early HIV treatment and care.

Outcomes along the HIV care continuum were associated with age. The lower percentages of younger people diagnosed, engaged in care, and on ART might reflect shorter duration of infection and less time for diagnosis. Further, the low percentage (13%) of persons aged 18–24 years achieving viral suppression is largely driven by the lower percentage diagnosed compared with other age groups. Although there were no significant differences in viral suppression by race/ethnicity, blacks had lower percentages than whites of being diagnosed and achieving viral suppression. Disparities along the HIV care continuum might reflect differences in access to and use of health care and treatment (12,13). Both targeted interventions and efforts to address underlying social determinants of health, such as increased access to health care and supportive services, could increase HIV diagnosis, engagement in care, ART, and viral suppression.

**Key Points**

- An estimated 1.2 million persons are living with human immunodeficiency virus (HIV) in the United States. Early HIV diagnosis, timely treatment, and HIV medical care can lead to viral suppression, which improves the health of persons living with HIV, increases survival, and prevents transmission to others.
- An estimated 30% of persons living with HIV in the United States were virally suppressed in 2011, compared with 26% in 2009.
- Persons aged 18–24 years, 25–34 years, and 35–44 years were less likely to have suppressed viral load compared with those aged ≥65 years.
- Among persons whose viral load was not suppressed, 20% had never been diagnosed with HIV, 66% were diagnosed but not engaged in HIV medical care, 4% were engaged in HIV medical care but not prescribed antiretroviral therapy (ART), and 10% were prescribed ART but had not achieved viral suppression.
- Increasing viral suppression will require increasing the percentages of persons with HIV who are aware of their infection (86% in 2011), engaged in HIV medical care (40% in 2011), and prescribed ART (37% in 2011).
- Additional information is available at http://www.cdc.gov/vitalsigns.

These findings are subject to limitations. First, linkage to care is based on persons newly diagnosed in 2011 using data from only 19 areas with complete laboratory reporting so might not be representative of the United States as a whole. Second, engagement in care might be underestimated, because only persons receiving HIV medical care during January–April, 2011 were considered engaged in care. Weighted population estimates of persons on ART and virally suppressed were only calculated among persons engaged in care, so an underestimate of engagement in care might also lead to underestimates of ART and viral suppression. Third, percentages of persons on ART might also reflect guidelines in effect in 2011 recommending ART initiation for persons with CD4+ count <500 cells/μL, which were revised in 2012 and now recommend treatment for all persons with HIV regardless of immune status. Fourth, viral suppression was defined based on laboratory results at last test and might not indicate durable viral suppression. Fifth, the number of persons living with HIV infection was estimated using NHSS data and was calculated for persons aged ≥13 years. Medical Monitoring Project data include persons aged ≥18 years, so persons aged 13–17 years are included in the denominator, but not the numerator, for estimates of percentages engaged in care, on ART, and virally suppressed. However, there were fewer than 5,000 persons aged 13–17 years living with diagnosed HIV in 2011, so this limitation is unlikely to substantially influence estimates.

State and local health departments, community-based organizations, and health care providers should work to reduce undiagnosed HIV infections and ensure that comprehensive services promoting linkage to, and engagement in, HIV medical care are available to all persons diagnosed with HIV. To support these efforts, CDC provides funding and technical assistance to reduce undiagnosed HIV infection, improve initial linkage and continued engagement in HIV medical care, increase viral suppression, and address disparities along the HIV care continuum. CDC is also working with state and local health departments to expand the use of HIV surveillance data in aggregate and on the individual level to improve engagement in HIV medical care and reduce viral load. The U.S. Department of Health and Human Services is supporting a range of projects to improve outcomes along the HIV care continuum, including research, Special Projects of National Significance, the Care and Prevention in the United States Demonstration Project, Partnerships for Care, Steps to Care,† and other innovative programs.

The findings in this report indicate that continued and intensified efforts are needed along the HIV care continuum. Only with success at each step in the continuum (i.e., diagnosing those with HIV, linking them to and engaging them in care, and ensuring they receive optimal treatment and prevention services) can the ultimate goals of improving health, reducing disparities, extending lives, and preventing further HIV transmission be achieved.

## Figures and Tables

**TABLE 1:** Estimated numbers and percentages of persons living with HIV, diagnosed with HIV, engaged in HIV medical care, prescribed ART, and virally suppressed by selected characteristics — National HIV Surveillance System and Medical Monitoring Project, United States and Puerto Rico, 2011

Characteristic	Total*		Diagnosed†		Engaged in care§		Prescribed ART¶		Virally suppressed**		
				
	No.	(%)	No.	(%)	No.	(%)	No.	(%)	No.	(%)	p value
**Total** ††	**1,201,100**	**(100)**	**1,032,800**	**(86)**	**478,433**	**(40)**	**441,661**	**(37)**	**361,764**	**(30)**	
**Sex** §§											
Male	920,900	(77)	784,900	(85)	352,523	(38)	326,061	(35)	271,358	(29)	Referent
Female	280,200	(23)	247,900	(88)	125,691	(45)	115,381	(41)	90,188	(32)	0.47
**Age group (yrs)**											
18–24¶¶	62,400	(5)	30,400	(49)	13,976	(22)	11,338	(18)	7,834	(13)	<0.01
25–34	165,500	(14)	122,500	(74)	55,934	(34)	49,105	(30)	37,667	(23)	<0.01
35–44	287,200	(24)	246,200	(86)	108,247	(38)	98,754	(34)	78,271	(27)	0.04
45–54	426,700	(36)	390,900	(92)	185,376	(43)	173,350	(41)	144,004	(34)	0.51
55–64	206,600	(17)	192,700	(93)	91,483	(44)	86,274	(42)	74,565	(36)	0.87
≥65	52,600	(4)	49,900	(95)	23,416	(45)	22,840	(43)	19,423	(37)	Referent
**Race/Ethnicity**											
Black/African American	491,100	(41)	417,500	(85)	195,159	(40)	178,237	(36)	137,740	(28)	0.55
Hispanic or Latino***	242,000	(20)	205,600	(85)	97,169	(40)	90,132	(37)	74,734	(31)	0.91
White	411,000	(34)	362,100	(88)	160,777	(39)	150,675	(37)	129,891	(32)	Referent
Other†††	57,000	(5)	47,600	(84)	25,328	(44)	22,617	(40)	19,399	(34)	—
**Transmission category** §§§											
Male-to-male sexual contact	647,700	(54)	543,900	(84)	246,545	(38)	227,015	(35)	191,190	(30)	Referent
Injection drug use											
Male	109,500	(9)	101,400	(93)	39,740	(36)	36,853	(34)	30,494	(28)	0.70
Female	70,100	(6)	65,600	(94)	32,703	(47)	29,706	(42)	23,784	(34)	0.45
Male-to-male sexual contact and injection drug use	64,800	(5)	60,300	(93)	30,817	(48)	28,532	(44)	22,789	(35)	0.31
Heterosexual contact¶¶¶											
Male	94,200	(8)	76,200	(81)	33,607	(36)	31,848	(34)	25,502	(27)	0.62
Female	209,700	(17)	180,600	(86)	90,989	(43)	83,676	(40)	65,072	(31)	0.70

**Abbreviations:** HIV = human immunodeficiency virus; ART = antiretroviral therapy.

* National HIV Surveillance System: Estimated number of HIV-infected persons who were aged ≥13 years and alive at year-end 2011. Numbers have been statistically adjusted to account for reporting delays and missing transmission category, but not for incomplete reporting.

† National HIV Surveillance System: Estimated number and percentage of HIV-infected persons who were aged ≥13 years, whose HIV infection had been diagnosed by year-end 2011. Numbers have been statistically adjusted to account for reporting delays and missing transmission category, but not for incomplete reporting.

§ Medical Monitoring Project: Estimated number and percentage of HIV-infected persons who were aged ≥18 years and received medical care during January–April 2011.

¶ Medical Monitoring Project: Estimated number and percentage of HIV-infected persons who were aged ≥18 years, received medical care during January–April 2011, and had documentation of ART prescription in the medical record.

** Medical Monitoring Project: Estimated number and percentage of HIV-infected persons who were aged ≥18 years, received medical care during January–April 2011, and whose most recent HIV viral load in preceding 12 months was undetectable or <200 copies/mL.

†† Numbers have been estimated and might not sum to total.

§§ Includes an estimated 6,674 persons whose self-identified sex differs from sex at birth.

¶¶ Estimated number of persons living with HIV and estimated number and percentage diagnosed includes persons aged 13–24 years.

*** Hispanics or Latinos can be of any race.

††† Includes American Indian/Alaska Native (N = 3,700 living with HIV; N = 3,000 diagnosed), Asian (N = 14,900 living with HIV; N = 11,600 diagnosed), Native Hawaiian/Other Pacific Islander (N = 1,200 living with HIV; N = 900 diagnosed), and multiple races (N = 37,200 living with HIV; N = 32,100 diagnosed).

§§§ Data have been statistically adjusted to account for missing transmission category. Transmission categories exclude persons whose HIV infection is attributed to hemophilia, blood transfusion, or perinatal exposure.

¶¶¶ Heterosexual contact with a person known to have, or to be at high risk for, HIV infection.

**TABLE 2:** Linkage to HIV medical care within 3 months after HIV diagnosis by selected characteristics—National HIV Surveillance System, 19 jurisdictions,* United States, 2011†

Characteristic	No. of HIV diagnoses	Linkage to care§	

		No.	(%)
**Total**	**15,449**	**12,333**	**(80)**
**Sex**			
Male	12,255	9,701	(79)
Female	3,194	2,632	(82)
**Age group (yrs)**			
13–24	3,445	2,528	(73)
25–34	4,482	3,509	(78)
35–44	3,381	2,804	(83)
45–54	2,826	2,381	(84)
≥55	1,315	1,111	(84)
**Race/Ethnicity**			
Black/African American	7,880	5,983	(76)
Hispanic or Latino¶	3,004	2,458	(82)
White	3,829	3,260	(85)
Other**	736	632	(86)
**Transmission category** ††			
Male-to-male sexual contact	9,943	7,874	(79)
Injection drug use			
Male	665	524	(79)
Female	455	371	(82)
Male-to-male sexual contact and injection drug use	436	344	(79)
Heterosexual contact§§			
Male	1,198	946	(79)
Female	2,732	2,256	(83)

**Abbreviation:** HIV = human immunodeficiency virus.

* The 19 jurisdictions were California (Los Angeles County and San Francisco only), Delaware, Georgia, Hawaii, Illinois, Indiana, Iowa, Louisiana, Michigan, Minnesota, Missouri, Nebraska, New Hampshire, New York, North Dakota, South Carolina, West Virginia, Wyoming, and the District of Columbia.

† Data include persons with a diagnosis of HIV infection regardless of stage of disease at diagnosis.

§ One or more CD4+ T-lymphocyte or viral load test within 3 months after HIV diagnosis.

¶ Hispanics or Latinos can be of any race.

** Includes American Indian/Alaska Native, Asian, Native Hawaiian/Other Pacific Islander, and multiple races.

†† Transmission categories exclude persons whose HIV infection is attributed to hemophilia, blood transfusion, or perinatal exposure.

§§ Heterosexual contact with a person known to have, or to be at high risk for, HIV infection.

## Footnotes

* The 19 jurisdictions were California (Los Angeles County and San Francisco only), Delaware, Georgia, Hawaii, Illinois, Indiana, Iowa, Louisiana, Michigan, Minnesota, Missouri, Nebraska, New Hampshire, New York, North Dakota, South Carolina, West Virginia, Wyoming, and the District of Columbia. Data from these areas represent 37% of data on diagnoses of HIV infection among persons aged ≥13 years during 2011 in the United States.

† Additional information on these programs available online: Special Projects of National Significance ( http://hab.hrsa.gov/abouthab/partfspns.html ), the Care and Prevention in the United States Demonstration Project ( http://www.cdc.gov/hiv/prevention/demonstration/capus ), Partnerships for Care ( http://www.cdc.gov/hiv/prevention/demonstration/p4c/index.html ), and STEPS to Care ( http://www.cdc.gov/hiv/pdf/policies_dhap_annualreport_2013.pdf ).

## References

[1] Samji H, Cescon A, Hogg RS (2013). Closing the gap: increases in life expectancy among treated HIV-positive individuals in the United States and Canada. PLoS One.

[2] Cohen MS, Chen YQ, McCauley M (2011). Prevention of HIV-1 infection with early antiretroviral therapy. N Engl J Med.

[3] CDC (2012). Estimated HIV incidence in the United States, 2007–2010.

[4] CDC (2014). Monitoring selected national HIV prevention and care objectives by using HIV surveillance data—United States and 6 dependent areas—2012.

[5] Blair JM, Fagan JL, Frazier EL (2014). Behavioral and clinical characteristics of persons receiving medical care for HIV infection—Medical Monitoring Project, United States, 2009. MMWR.

[6] Oehlert GW (1992). A note on the delta method. American Statistician.

[7] Branson BM, Handsfield HH, Lampe MA (2006). Revised recommendations for HIV testing of adults, adolescents, and pregnant women in health-care settings. MMWR.

[8] Moyer VA, U.S. Preventive Services Task Force (2013). Screening for HIV: U.S. Preventive Services Task Force recommendation statement. Ann Intern Med.

[9] CDC (2014). Compendium of evidence based interventions and best practices for HIV prevention.

[10] Thompson MA, Mugavero MJ, Amico KR (2012). Guidelines for improving entry into and retention in care and antiretroviral adherence for persons with HIV: evidence-based recommendations from an International Association of Physicians in AIDS Care panel. Ann Intern Med.

[11] Panel on Antiretroviral Guidelines for Adults and Adolescents (2014). Guidelines for the use of antiretroviral agents in HIV-1-infected adults and adolescents.

[12] DeNavas-Walt C, Proctor BD, Smith JC (2013). Income, poverty, and health insurance coverage in the United States: 2012.

[13] Wohl AR, Galvan FH, Myers HF (2011). Do social support, stress, disclosure and stigma influence retention in HIV care for Latino and African American men who have sex with men and women?. AIDS Behav.

